# Regional Differences in Correlates of Daily Walking among Middle Age and Older Australian Rural Adults: Implications for Health Promotion

**DOI:** 10.3390/ijerph13010116

**Published:** 2016-01-08

**Authors:** James Dollman, Melissa Hull, Nicole Lewis, Suzanne Carroll, Dorota Zarnowiecki

**Affiliations:** 1 Alliance for Research in Exercise, Nutrition and Activity, Sansom Institute for Health Research, University of South Australia, CEA-14, GPO Box 2471, Adelaide 5001, SA, Australia; melissa.hull@mymail.unisa.edu.au (M.H.); nlewis@yorkeandmidnorth.com.au (N.L.); 2 Spatial Epidemiology and Evaluation Research Group, Centre for Population Health Research, Sansom Institute for Health Research, University of South Australia, IPC CWE-48, GPO Box 2471, Adelaide 5001, SA, Australia; suzanne.carroll@unisa.edu.au; 3 School of Health Sciences, Sansom Institute for Health Research, University of South Australia, Adelaide 5001, SA, Australia; dorota.zarnowiecki@unisa.edu.au

**Keywords:** physical activity, walking behavior, correlates, rural communities

## Abstract

Rural Australians are less physically active than their metropolitan counterparts, and yet very little is known of the candidate intervention targets for promoting physical activity in rural populations. As rural regions are economically, socially and environmentally diverse, drivers of regular physical activity are likely to vary between regions. This study explored the region-specific correlates of daily walking among middle age and older adults in rural regions with contrasting dominant primary industries. Participants were recruited through print and electronic media, primary care settings and community organisations. Pedometers were worn by 153 adults for at least four days, including a weekend day. A questionnaire identified potential intra-personal, social and environmental correlates of physical activity, according to a social ecological framework. Regression modelling identified independent correlates of daily walking separately in the two study regions. In one region, there were independent correlates of walking from all levels of the social ecological framework. In the other region, significant correlates of daily walking were almost all demographic (age, education and marital status). Participants living alone were less likely to be physically active regardless of region. This study highlights the importance of considering region-specific factors when designing strategies for promoting regular walking among rural adults.

## 1. Introduction

Rural Australians are burdened by earlier mortality, and higher levels of chronic disease and health risk factors relative to urban Australians [1]. There is evidence that rural adults are relatively less active than their urban counterparts [2] which may in part explain their poorer health outcomes.

Aetiological relationships between rural residence and physical activity are poorly understood, but compositional and contextual factors are likely to be important. Rural residents are generally older [3] and have lower levels of education and income [4], all related to lower habitual physical activity in the general adult population [5]. Environmental characteristics of rural regions have also been linked to relatively low physical activity, including weaker social support [6], poorer accessibility and availability of places to be physically active, extreme weather without built mitigation such as shading and fresh drinking water, heavy vehicle traffic on major roads, low quality footpaths, inadequate street lighting in towns and larger distances to destinations such as shops and services [7,8,9,10,11].

Previous studies of rural health and health behaviours have largely adopted a two dimensional perspective (metropolitan *vs.* rural) to examine differences in behaviours or behavioural determinants by location of residence [6]. These studies have reported “unique” aspects of rurality that should be considered when planning health promotion policies and practices in rural settings.

However, an implicit assumption in these study designs is that rural regions are relatively homogeneous in regard to compositional and contextual influences on health-related behaviours. This assumption disregards inter-region variability in demographic, climatic, geographic and economic factors that can create region-specific settings for health-related behaviours [12]. For instance, the dominant primary industries of a region impact on factors such as population density and distribution, average age and income, gender roles, physical demands of occupation and daily time use patterns, all of which may impact on physical activity options and preferences in the community.

There is growing support for setting-specific health promotion driven by community development and capacity building based on thorough community consultation [13]. This approach takes account of important regional differences by allowing responses at all levels of the social ecological framework [14] to evolve from planning to action within each regional or neighbourhood milieu. However, to the authors’ knowledge, there is no published evidence to support development of physical activity promotion strategies within the context of local rural communities.

Walking is a logical target for physical activity promotion as it is free, readily accessible, not dependent on training or equipment [15], and accurately measured using pedometers [16]. A recent review by Kelly and colleagues [15] found that walking markedly reduces all-cause mortality, independent of other physical activity, and that the largest reduction in all-cause mortality is evident among those who move from no walking to some regular walking. Of concern, ongoing health surveillance data reveal that regular walking among South Australian adults is less likely among older adults and those residing in rural and remote regions of the state [17].

The aim of this study was to identify correlates of walking, represented by pedometer steps, among insufficiently active middle age and older adult residents of two South Australian rural regions that differ by primary industry profile. By identifying the correlates of walking in separate regions, the study is the first to explore the extent to which physical activity promotion in rural Australia should adopt a community development framework rather than a “one size fits all” approach.

## 2. Experimental Section

### 2.1. Recruitment

This analysis was conducted on baseline data from a six week walking intervention in 2011 among insufficiently active adults in rural South Australia. Participants were men and women from two rural regions, the Riverland and Yorke Peninsula, that differ in terms of dominant primary industries but are matched as closely as possible on demographic profile. The leading primary industry in the Riverland region is irrigated horticulture and viticulture, and includes grape growing, citrus production, beverage manufacturing, and dryland farming (crop and pasture production). The dominant employment sector within the region is fruit and tree nut growing (14.0%) [18]. The Yorke Peninsula centres on primary production and processing, with major industries including grain and livestock production, fishing and aquaculture, and mining and mineral processing. The dominant employment sector within the region is sheep, beef cattle and grain farming (24.4%) [19].

Participants were recruited through local newspaper advertisements, local radio, advertisement in school newsletters and local businesses, sporting and community clubs. Additional face to face recruitment was conducted in local shopping centres. The pool of potential volunteers was screened over the telephone to identify those who were permanent residents of the target regions, over 40 years of age, insufficiently active according to self-report, and free of conditions that would limit engagement in a walking program. This age group was targeted as recent state-wide physical activity surveys have identified lower levels of habitual physical activity among those aged 40 years and older [17]. The following single item was used to identify current activity levels: “In the past month, on how many days have you done a total of 30 min or more of physical activity, which was enough to raise your breathing rate? This may include sport, exercise, and brisk walking or cycling for recreation or to get to and from places, but should not include housework or physical activity that may be part of your job” [20]. An open-response scale was used, with valid responses ranging from 0 to 31 days. Based on the extant national guidelines [21], responses of less than 20 days were deemed to represent insufficient physical activity (*i.e.*, an average of less than 5 days per week). Eligible participants were further screened using Phase 1 of the Sports Medicine Australia Adult pre-exercise screening tool [22] designed for light-moderate physical activity. Those with elevated risk were required to obtain medical clearance before inclusion in the study (*n* = 19). The study protocol was approved by the Human Research Ethics Committee of the University of South Australia (Project number: 0000025069).

### 2.2. Physical Activity; Daily Steps

At baseline, participants wore a sealed New Lifestyles (NL) 1000^®^ pedometer for seven days, during which they diarised time of pedometer removal on each day. Previous studies have shown that the piezo-electric mechanism of the NL series is superior to spring-levered instruments for accurately recording steps in obese individuals [23], of particular relevance to the study population given the disproportionately high rates of adult obesity in rural Australia [24]. The NL 1000 also has the advantage over other pedometers of storing daily step counts for the previous 7 days, avoiding the burden on participants of logging steps each evening and the potential measurement bias associated with this procedure [16]. The likelihood of data loss through accidental or deliberate tampering is negligible due to the complex setting sequences of the NL 1000. This was further reduced by sealing the unit. The data were retrieved from the pedometer memory by the research team after the collection of the pedometer from the participants. Daily records were excluded if the pedometer was worn for less than 10 h or <1000 steps were recorded [25]. There were 216 non-compliant days overall. The average of daily steps was calculated for participants with a minimum of 4 compliant days, including one weekend day, in accord with published recommendations [26]. For analytical purposes, step categories were formed according to cut-points for healthy adults recommended by Tudor-Locke and Bassett, Jr [27]: <5000 steps·day^−1^ (inactive); 5000 to 7499 steps·day^−1^ (low active); 7500 to 9999 steps·day^−1^ (somewhat active); 10,000 to 12,499 steps·day^−1^ (active); and >12,500 steps·day^−1^ (highly active).

### 2.3. Anthropometry

Weight and stretch stature were measured according to the protocols of the International Society for the Advancement of Kinanthropometry (ISAK) [28]. Body mass index (BMI) was calculated as weight (kg) divided by stature (m) squared. Weight status was defined as normal (<25 kg·m^−2^), overweight (25–29.99 kg·m^−2^) and obese (≥30 kg·m^−2^). There were no underweight participants.

### 2.4. Correlates Questionnaire

The questionnaire assessed domains according to the social ecological framework that have demonstrated associations with adult physical activity behaviours [14] (see Table 1): demographic; biological; psychological; social; and environmental. Questions used were adopted from existing questionnaires [29,30,31,32,33,34,35]. Response categories for the questionnaire were 5-point Likert scales, with exact options varying between domains. All items were coded positively whereby a higher score indicated a more positive score/higher agreement or confidence. The compiled correlates questionnaire and specific response options used in this study are detailed in Supplementary material.

Multiple items relating to the same concept (e.g., self-efficacy) were averaged to form a representative score and assessed for internal consistency using the Cronbach α statistic (Supplementary material). Other single items were entered into models individually.

**Table 1:** Sample descriptive characteristics by region.

Variable	Riverland (*n = 67*)	Yorke Peninsula (*n = 86*)
Demographics		
Age (years) ^#^	60.19 (8.80)	59.48 (9.02)
Sex: female *n* (%)	39 (58.2)	63 (73.3)
Highest education *n* (%)		
Some/completed primary school	3 (4.5)	2 (2.3)
Some high school	28 (40.8)	35 (40.7)
Completed high school	7 (1.5)	9 (10.5)
Trade or diploma	12 (17.9)	15 (17.4)
University degree or higher	14 (20.9)	23 (25.6)
Marital status: single *n* (%)	16 (23.9)	27 (31.4)
Manage on income ^#^	3.66 (0.91)	3.85 (0.87)
Work status: unemployed/not in labour-force *n* (%)	20 (29.9)	35 (40.7)
BMI ^#^ (kg/m^2^)	30.89 (5.85)	30.59 (4.94)
Weight category *n* (%):		
Normal weight (<25 kg/m^2^)	7 (10.4)	8 (9.3)
Overweight/obese(≥25 kg/m^2^)	6 (89.6)	78 (90.7)
**Physical activity**		
Daily steps ^#^	8429.13 (3733.21)	7506.47 (2767.71)
Activity category *n* (%):		
Inactive (<5000 steps)	11 (16.4)	17 (19.8)
Low active (5000–7499)	22 (32.8)	32 (37.2)
Somewhat active (7500–9999)	14 (20.9)	20 (23.3)
Active (10,000–12,499)	10 (14.9)	14 (16.3)
Highly active (>12,500)	10 (19.9)	3 (3.5)
**Biological**		
General health ^#^	2.90 (0.89)	2.97 (0.85)
**Psychological**		
Motivation ^#^	3.83 (0.55)	3.90 (0.58)
Barriers self-efficacy ^#^	3.26 (1.04)	3.03 (0.93)
Relapse self-efficacy ^#^	3.20 (1.03)	3.11 (0.85)
Already active ^#^	2.82 (1.30)	2.71 (1.10)
Bullet-proof ^#^	1.82 (1.07)	1.90 (0.92)
Need a health scare ^#^	2.39 (1.18)	2.30 (1.15)
Physical activity important ^#^	4.29 (0.65)	4.07 (0.72) *
**Social**		
Others active in neighbourhood ^#^	3.27 (1.02)	3.27 (1.03)
Need for support ^#^	3.52 (0.76)	3.56 (0.84)
**Environmental**		
Pleasant community ^#^	1.60 (0.87)	1.67 (0.89)
Safety ^#^	2.04 (1.00)	2.21 (1.10)
Walkability ^#^	3.51 (1.13)	3.12 (0.87) *

Notes: * *p* < 0.05; ^#^ presented as mean (SD); BMI, Body mass index.

### 2.5. Statistical Analyses

Descriptive statistics are presented as mean (±standard deviation) for continuous variables and frequencies for categorical variables. Participant responses from the two regions were compared on measured variables using *t* tests or Chi square as appropriate. Simple regression models of daily steps explored interactions of region (Riverland *vs*. Yorke Peninsula) with each potential correlate variable in turn. An alpha level of ≤0.2 was set to infer significance in these exploratory models [36]. As 10 of 20 possible interactions were significant, modelling was performed separately in each region. Stratified by region, ordinal logistic regression was performed including all independent variables, with step category as the dependent variable. Finally, the most parsimonious models of step category in each region were established using stepwise backward elimination with alpha set at *p* < 0.05. All analyses were conducted using STATA (version 12.0, Statacorp, College Station, TX, USA). The STATA command used for this analysis automatically screens for multicollinearity (set at VIF > 10); because of collinearity between barriers self-efficacy and relapse self-efficacy, regression modelling avoided the simultaneous inclusion of these two variables.

## 3. Results

### 3.1. Sample Characteristics: Comparisons of Regions

There were no statistically significant differences in age, gender, highest education level and BMI for those with (*n* = 175) and those without (*n* = 9) compliant pedometer data. Further sample loss due to incomplete data resulted in 153 participants (Riverland *n* = 67, Yorke Peninsula *n* = 86) with data available for analysis. In the whole analysis sample, 66.7% were female (58.2% in the Riverland and 73.3% in the Yorke Peninsula) and 90.2% were overweight or obese (89.6% in the Riverland and 90.7% in the Yorke Peninsula). There were no statistically significant differences between region samples for demographic characteristics (see Table 1). However, it should be noted that the proportion of participants who were female was borderline statistically significantly different between regions (Pearson’s Chi^2^ *p* = 0.05).

Compared with Australian Bureau of Statistics (ABS) Census data for adults 35 years and over within the relevant regions (40 years and over for age and sex data) [37], Riverland participants in the current study were statistically significantly: more likely to be in the 60–70 years age group (ABS, 22.7%; current study, 44.8%, *p* < 0.0001); more likely to have a university degree (ABS, 7.0%; current study, 20.9%, *p* < 0.0001); and more likely to be full- or part-time employed (ABS, 51.4%; current study, 70.1%, *p* < 0.01). Similarly, Yorke Peninsula participants in the current study were statistically significantly: more likely to be in the 60–70 years age group (ABS, 26.6%, current study, 40.7%, *p* < 0.01); more likely to be female (ABS, 51.9%, current study, 73.3%, *p* < 0.0001); more likely to have a university degree (ABS, 5.6%; current study, 27.7%, *p* < 0.0001); and more likely to be full- or part-time employed (ABS, 40.2%; current study, 59.3%, *p* < 0.001).

Regarding hypothesised correlates of walking steps, participants in the Yorke Peninsula reported statistically significant lower levels of “physical activity important” and “walkability” compared to their Riverland counterparts. There were no other statistically significant differences in reporting of hypothesised correlates, nor was there a statistically significant difference in step counts or categories between regions.

### 3.2. Correlates of Walking (Step Category)

In the Riverland sample, there were independent correlates of step category from all levels of the social ecological framework represented in the analysis (see Table 2). General health, “need for support”, safety and “pleasant community” were significant in both full and parsimonious models, which explained 31% and 28% of explained variance in step category, respectively. In the Yorke Peninsula, demographic variables (age, education and marital status) were the principal contributors to the full and parsimonious models of step categories, which explained 22% and 8% of explained variance in step category, respectively. Marital status was the only correlate of walking step category in all models, with living alone associated with less walking. 

**Table 2:** Full and parsimonious ordinal logistic regression model results: odds ratios (OR) and 95% CI for included independent variables in predicting pedometer step categories.

Correlate	Riverland (*n* = 67)		Yorke Peninsula (*n* = 86)	
	Full Model	SW Model	Full Model	SW Model
	OR (95% CI)	OR (95% CI)	OR (95% CI)	OR (95% CI)
**Demographic**				
Age (years)	0.93 (0.85–1.02)	0.90 (0.83–0.97) **	0.88 (0.82–0.95) ***	0.89 (0.85–0.94) ****
**Sex**				
Female	2.10 (0.66–6.64)	-	1.12 (0.40–3.15)	-
Male (referent)	-	-	-	-
**Education**	0.71 (0.45–1.12)	-	0.64 (0.45–0.91) *	0.71 (0.5347–0.95) *
**Marital status:**				
Single	0.16 (0.04–0.64) **	0.60 (0.42–0.84) **	0.17 (0.05–0.52) **	0.41 (0.17–0.99) *
Married/de facto (referent)	-	-	-	-
Income	1.66 (0.83–3.32)	1.88 (1.07–3.32) *	1.08 (0.59–2.00)	-
**Job status:**				
Unemployed/not in labour force	0.34 (0.07–1.64)	-	0.42 (0.13–1.30)	-
Full- or part-time employed (referent)	-	-	-	-
BMI (kg/m^2^)	1.00 (0.89–1.13)	-	0.99 (0.91–1.09)	-
General health	4.00 (1.68–9.50) **	2.97 (1.36–6.48) **	1.18 (0.66–2.12)	-
**Psychological**				
Motivation	0.83 (0.25–2.74)	-	0.47 (0.20–1.11)	-
Barriers self-efficacy	0.98 (0.51–1.88)	-	0.63 (0.34–1.15)	-
Relapse self-efficacy	0.63 (0.32–1.23)	-	1.10 (0.59–2.05)	-
Already active	1.41 (0.72–2.72)	1.93 (1.18–3.15) **	1.55 (0.86–2.80)	-
“Bullet-proof”	1.76 (0.78–3.98)	-	0.96 (0.55–1.69)	-
“Need a health scare”	1.84 (0.95–3.53)	2.33 (1.39–3.90) **	1.36 (0.92–2.02)	-
Physical activity important	1.25 (0.50–3.17)	-	3.46 (1.49–8.03) **	-
**Social**				
Others active	0.93 (0.47–1.81)	-	0.69 (0.38–1.24)	-
Need for support	0.33 (0.14–0.78) *	0.47 (0.23–0.94) *	0.78 (0.38–1.61)	-
**Environmental**				
Pleasant community	5.85 (2.01–16.99) ***	2.31 (1.20–4.44) **	0.53 (0.28–1.00)	0.62 (0.40–0.97) *
Safety	0.40 (0.21–0.78) **	0.47 (0.27–0.82) **	0.85 (0.51–1.43)	-
Walkability	2.45 (1.08–5.55) *	-	1.39 (0.71–2.69)	-
*Model pseudo R^2^*	*0.31*	*0.28*	*0.22*	*0.08*

Notes: BMI, body mass index; * *p* ≤0.05, ** *p* ≤ 0.01, *** *p* ≤ 0.001, **** *p* ≤ 0.0001, SW = Stepwise.

## 4. Discussion

Walking has been described as the “nearest activity to perfect exercise” [38] (p. 306) and walking is a key target in population-wide physical activity promotion [39] due to its association with reduced risk of all-cause mortality [15]. In rural Australia, where chronic disease is relatively prevalent, surprisingly little is known about the factors that shape walking behaviour. In this study, utility of the social ecological framework for understanding physical activity behaviours was somewhat supported in one region, the Riverland, with influences from all levels of this framework contributing to prediction models of daily step categories. In the Yorke Peninsula sample, much less variance in the outcome was explained overall, largely by demographic variables. In reference to the aim of the study, there were clear differences in the pattern of correlates between regions.

The results of this study suggest the need to explicate influences on walking behaviour “from the ground up” in each rural region, and that a “one size fits all” approach to physical activity promotion in rural Australia may lead to lower overall success rates. For instance, efforts to promote regular walking in adult residents of the Yorke Peninsula are more likely be successful if older residents are targeted. On the other hand, Riverland adults are likely to benefit from a focus on improving safety for walking, providing options that better meet the needs of those with relatively poor general health, and shifting attitudes towards personal health from a reactive to a proactive perspective. In both regions, strategies that particularly support those living alone are warranted.

In the Yorke Peninsula, participants with higher education were less likely to be in the active step categories. This conflicts with much of the literature that reports a positive association of socioeconomic status and self-reported habitual physical activity among Australian adults [17]. It is feasible that in our study those with relatively high education status were more likely to be employed in desk-based office jobs that are relatively sedentary, while those with lower education are more likely to work on farms or in other active occupations [40]. Unfortunately the measurement of daily physical activity by pedometry does not allow for measurement time frames to be segmented into work and non-work time.

Notably, walkability as defined in this study was not associated with walking in parsimonious models in either study region. This observation is in accord with a recent Australian study that showed stronger associations of the perceived physical activity environment (that included elements of walkability) and leisure-time physical activity among urban compared with rural middle-to-older aged adults [11]. Nevertheless, it must be acknowledged that a thorough assessment of walkability should focus on walking for different purposes (e.g., recreation, transport and occupation) separately, and should include details of the route and destinations. Given the size of the questionnaire used in the current study, the inclusion of such detail would have added considerably, and perhaps prohibitively, to participant burden. Another plausible explanation may lie in the structure of the data. As walkability was generally rated highly by study participants, there may have been limited variability in the scores on this domain to explain variance in the dependent variable, despite the fact that walkability may still be ecologically important for walking behaviours.

A number of strengths and limitations of this study warrant acknowledgment. Daily walking was assessed using the best available step measurement in a predominantly inactive and overweight adult population [23]. Further, the data storage capacity of the pedometer allows covert step recording that minimises reactivity associated with participants recording daily steps, which can be as high as ~15% [25]. Nevertheless, awareness among participants that their physical activity was being monitored can still lead to altered behaviour in the monitoring period [16]. While impossible to quantify, it is likely that this effect would be similar in the two study regions and would negligibly distort statistical modelling.

True region-specific correlates of regular walking may have been masked by the convenience sampling which led to an under-representation of male participants and an over-representation of overweight and obese participants. The targeting of insufficiently active people for recruitment into a walking intervention may have contributed to the different model structures in the two regions given that a higher proportion of Yorke Peninsula residents were insufficiently active. It is also interesting to note that 33% of participants in the Riverland and 30% in the Yorke Peninsula agreed or strongly agreed that they were “already active” and yet had volunteered to participate in an intervention to promote regular walking. This suggests that the attraction of many rural adults to physical activity promotion initiatives might be driven by motivators other than health improvement that were not measured in the current study. Nevertheless, the overall findings of this study are important from the health promotion perspective as over 50% of participants in both regions were “low active” or “inactive”, among whom the relatively largest health gains are associated with an achievable increase in regular walking of up to 120 min·wk^−1^ [15].

The focus of this study was on correlates of overall daily walking. This research could be extended to identify correlates of: walking for different reasons, such as recreation, transport and occupation; types of physical activity other than walking; and sedentary behaviours such as prolonged sitting which are independently associated with several health outcomes [41]. Finally, the research should be scaled up to include more than two rural regions, representing a wider diversity of primary industries, to confirm the regional specificity of correlates of walking seen in this study.

Arising from their discussions with health care professionals in rural Australia, Allan, Ball and Alston [42] wrote of the idiosyncratic “personalities” of small rural townships. Others have highlighted the uniqueness of rural communities and regions in relation to health. Ricketts [12] (p. 44) noted that urban–rural comparisons are “plagued by the problem of aggregation of widely divergent nonmetropolitan populations…” while there are “regional patterns of rural disadvantage.” In their scoping review of sustainability in community-based obesity prevention, Whelan and colleagues [43] highlighted the critical importance of engaging communities to take stronger control over the determinants of health that are relevant to their context. By considering the unique attributes, assets and interests of individual communities, initiatives are likely to achieve better “buy in” by promoting a strong sense of community ownership. One of the key challenges is to address the lack of confidence among local governments and policy makers to adopt a broad health promotion agenda at the local level [44] by providing the best available research evidence to guide community-level decision making for health promotion initiatives [43].

## 5. Conclusions

From a public health perspective, higher levels of habitual walking will effectively reduce all-cause mortality [15]. The results of this study indicate that meeting the challenge of relatively poor health among rural Australians through physical activity promotion will require further investigation of region-specific approaches that take full account of local resources and population attributes.

## Supplementary Files

Supplementary File 1
