# Supplementary material for: Regional Differences in Correlates of Daily Walking among Middle Age and Older Australian Rural Adults: Implications for Health Promotion
Source: Int J Environ Res Public Health. 2016 Jan 8;13(1):116. doi: 10.3390/ijerph13010116 (PMC4730507; doi:10.3390/ijerph13010116)
Supplement: Supplementary File 1 [file ijerph-13-00116-s001.pdf]

Supplementary Materials: Regional Differences in Correlates of Daily Walking among Middle Age and Older Australian Rural Adults: Implications for Health Promotion

James Dollman, Melissa Hull, Nicole Lewis, Suzanne Carroll and Dorota Zarnowiecki

Table S1. Individual items and factors (including Cronbach α's) from the physical activity correlates questionnaire.

Questionnaire Item	Factors	Response Option	Cronbach α
	Demographic		
Education	*What is the highest level of education you have completed?*	Never attended school; Some primary school; Completed primary school; Some high school; Completed high school; Trade or trade certificate or diploma; University or tertiary institute degree; Higher University degree (e.g., Postgraduate diploma, Masters, PhD)	n/a
Work Status	*Which of the following best describes your main daily activities and/or responsibilities?*	Employed full-time; Employed part-time; Self-employed; Unemployed; Home/duties and/or raising children full-time; Student; Retired; Unable to work; Don't know/unsure	n/a
Marital status	*Which of the following best describes your current marital status?*	Married; Living with a partner; Separated; Divorced; Widowed; Never married; Don't know/unsure	n/a
Income	*Which of the following best describes how you manage on the income you have available?*	It is impossible; It is difficult all the time; It is difficult some of the time; It is not too bad; It is easy; Don't know/unsure	n/a
	Biological		
General health	*What would you say is your general health?*	Poor; Fair; Good; Very good; Excellent	n/a
	Psychological		
Motivation [29]	*I'm good at keeping promises, especially the ones I make to myself.*<br>*When I take on a difficult job, I make a point of sticking with it until it's completed.*<br>*I have a lot of self-motivation.*<br>*I'm good at making decisions and standing by them.*<br>*I work harder than most of my friends.*<br>*Sometimes I push myself harder than I should.*<br>*I like to take on jobs that challenge me.*<br>*Whenever I reach a goal, I set a higher one.*<br>*I can persist in spite of failure.*<br>*I have a strong desire to achieve.*	Very unlike me; Unlike me; Neither like or unlike me; Somewhat like me; Very much like me	0.75

Table S1. *Cont.*

Questionnaire Item	Factors	Response Option	Cronbach α
Barriers self-efficacy [30]	*How confident are you that you could do the following if you wanted to:*<br>*Go for a walk for exercise regularly for the next 6 months;*<br>*Be physically active in your leisure time even when tired;*<br>*Be physically active in your leisure time even when in a bad mood;*<br>*Be physically active in your leisure time even when you feel you don't have time;*<br>*Be physically active in your leisure time even if you have no one to be active with.*	Not at all confident; Somewhat confident; Moderately confident; Very confident; Completely confident	0.80
Relapse self-efficacy [31]	*If I have started to do physical activity regularly, I am confident that I can maintain it, even if I don't see immediate results.*<br>*I am confident that I am able to resume regular physical activities, even if I had failed to maintain them a couple of times.*<br>*I am confident that I can resume my physical activity, even when feeling weak after an illness.*<br>*If I have started to do physical activity regularly, I am confident that I can maintain it, even if I don't see immediate results.*	Not at all confident; Somewhat confident; Moderately confident; Very confident; Completely confident	0.93
Health beliefs [32]	No factor formed		n/a
Already active	*I am doing sufficient regular exercise for good health.*	Strongly disagree; Somewhat disagree; Neutral; Somewhat agree; Strongly agree	
Bullet-proof	*I take the attitude that I am "bullet-proof", so there is no need to look after my health.*		
Need a health scare	*I need a health scare before I am inclined to change my exercise behaviour.*		
Physical activity Important	*Being physically active for my health is important to me.*		

Table S1. *Cont.*

Questionnaire Item	Factors	Response Option	Cronbach α
	Social		
Need for support [33]	*If I had someone like a friend or family member to exercise with, chances are that I would exercise more.*<br>*I would maintain my regular exercise if my friends encouraged me to exercise.*<br>*I will maintain my regular exercise if my friends encourage me to exercise.*	Strongly disagree; Somewhat disagree; Neutral; Somewhat agree; Strongly agree	0.82
Others active	*A lot of people in my neighbourhood are physically active.*	Strongly disagree; Somewhat disagree; Neutral; Somewhat agree; Strongly agree	n/a
	Environmental		
Walkability [34]	*Many shops, stores, markets or other places to buy things I need are within easy walking distance.*<br>*There are footpaths on most of the streets in my neighbourhood.*<br>*My neighbourhood offers many opportunities to be physically active.*<br>*My neighbourhood has several free or low cost recreational facilities, such as parks, walking trails, bike paths, recreation centres, playgrounds, public swimming pools.*<br>*There are many interesting things to look at while walking in my neighbourhood.*<br>*There are many places to be physically active in my community not including streets (e.g., parks, trails, playgrounds)*	Strongly disagree; Somewhat disagree; Neutral; Somewhat agree; Strongly agree	0.88
Pleasant community [33]	*Overall, how would you rate your community as a place to be physically active?*	Very pleasant; Somewhat pleasant; Not very pleasant; Not at all pleasant	n/a
Neighbourhood risk [35]	*The crime rate in my neighbourhood makes it difficult or unpleasant to go on walks at nights.*<br>*Traffic on the streets or roads that it makes it difficult or unpleasant to walk in my neighbourhood.*	Strongly disagree; Somewhat disagree; Neutral; Somewhat agree; Strongly agree	0.71

© 2016 by the authors; licensee MDPI, Basel, Switzerland. This article is an open access article distributed under the terms and conditions of the Creative Commons by Attribution (CC-BY) license (http://creativecommons.org/licenses/by/4.0/).
